# Racial Group Membership Is Associated to Gaze-Mediated Orienting in Italy

**DOI:** 10.1371/journal.pone.0025608

**Published:** 2011-10-04

**Authors:** Giulia Pavan, Mario Dalmaso, Giovanni Galfano, Luigi Castelli

**Affiliations:** Dipartimento di Psicologia dello Sviluppo e della Socializzazione, Università di Padova, Padova, Italy; French National Centre for Scientific Research, France

## Abstract

Viewing a face with averted gaze results in a spatial shift of attention in the corresponding direction, a phenomenon defined as gaze-mediated orienting. In the present paper, we investigated whether this effect is influenced by social factors. Across three experiments, White and Black participants were presented with faces of White and Black individuals. A modified spatial cueing paradigm was used in which a peripheral target stimulus requiring a discrimination response was preceded by a noninformative gaze cue. Results showed that Black participants shifted attention to the averted gaze of both ingroup and outgroup faces, whereas White participants selectively shifted attention only in response to individuals of their same group. Interestingly, the modulatory effect of social factors was context-dependent and emerged only when group membership was situationally salient to participants. It was hypothesized that differences in the relative social status of the two groups might account for the observed asymmetry between White and Black participants. A final experiment ruled out an alternative explanation based on differences in perceptual familiarity with the face stimuli. Overall, these findings strengthen the idea that gaze-mediated orienting is a socially-connoted phenomenon.

## Introduction

People's gaze conveys several information to an observer. Humans and other animal species have developed the ability to rapidly orient attention towards the location where the gaze of another is directed in order to detect potentially relevant events in the environment [see 1 for a review]. This ability is particularly relevant in everyday life in that, by identifying others' focus of attention, humans can draw inferences about the intentions and future behaviors of others. Given this significance, a great bulk of research has been dedicated to the eyes and the message they convey and, in recent years, several studies have focused on the mechanism known as gaze-mediated orienting of attention [see 2 for a review]. In the classic paradigm used to study this phenomenon, a face is presented in the center of the screen first with direct gaze, and then with averted gaze thus conveying the impression of eye movement [3]. Subsequently, a target appears in a location which is either congruent or incongruent with gaze direction. Results consistently show that, even though gaze direction is not informative as to target location, participants are faster to process the target on congruent than on incongruent trials. This effect is interpreted as reflecting an automatic shift of attention in the direction signaled by the gaze. Thus, processing a target appearing in that location requires less time as compared to a target appearing in the opposite location (i.e., cueing effect: reaction time advantage for spatially congruent over spatially incongruent trials). Even though previous studies characterized this phenomenon as being reflexive and involuntary [4], more recent work has shown that social factors can interact with gaze-mediated orienting of attention, thus challenging its unconditioned automaticity. For instance, a flourishing literature investigated the role of emotional expressions bored by the cueing face in modulating gaze-mediated orienting of attention showing that certain kinds of emotions (e.g., fear) may enhance the cueing effect [e.g.], [ 5], [ 6; see 7 for a review]. Another study has recently investigated the role of dominance [8]. To this aim, faces of both males and females have been modified in a way to accentuate their feminine or masculine traits and participants have been presented with these faces in a modified spatial cueing paradigm. Faces characterized by masculine traits are known to be perceived as more dominant whereas faces characterized by feminine traits are perceived as subordinates [e.g.], [ 9, 10]. The results demonstrated that masculinized faces triggered a greater cueing effect as compared to feminized faces [8]. The research described above shows that the physical features of a face and the message they convey can influence the orienting process. However, so far, only few studies have addressed the influence of the social relationship between the participant and the face presented on the screen in modulating the cueing effect. For instance, it was found that when pictures of familiar faces are used as stimuli, the cueing effect is enhanced, even though only for female participants [11]. Moreover, a study conducted with macaques which investigated the role of hierarchical relationships within a group demonstrated that subordinate animals show a similar cueing effect towards both same status and higher status conspecifics, whereas dominant animals show a larger cueing effect for same status than for subordinate conspecifics [12], confirming an earlier idea proposed by Chance (1967) [13]. Intra-group processes thus seem to modulate the cueing effect among non human primates.

To our knowledge, no previous work has systematically addressed the impact of intergroup processes on gaze cueing. In the present research we explored this issue by focusing on racial group membership, comparing the responses of Black and White people when presented with Black and White faces. There is strong evidence that the interaction between the racial group membership of the perceiver and that of the person perceived may deeply shape human basic cognitive processes. For instance, it has been shown that empathic sensorimotor resonance when observing the pain of other individuals is present for racial ingroup but not outgroup members [14]. Moreover, recent evidence has shown that, in the context of different racial groups, the perception of touch is enhanced for faces belonging to the ingroup [15]. As regards the gaze cueing phenomenon, one possibility is that this effect is boosted by shared group membership. In other words, according to this hypothesis, White participants should show a magnified gaze cueing effect when viewing a White face with respect to a Black face. In the same vein, Black participants should reveal a stronger gaze cueing effect for Black faces with respect to White faces. There is however a second possibility based on the existing asymmetries between Black and White individuals. Indeed, at least in most Western societies, White people can be considered as the majority group, in that they are more likely associated to positive evaluations [16], [17], positive stereotypes [18], [19], and corresponding higher positions within the society as compared to Black people. This is true also in the social environment where the present research was conducted, namely the Italian context. Indeed, Black people in Italy are a stigmatized minority group, not yet fully integrated in the society [20] and still the target of prejudiced attitudes [21]. A recent survey from the Italian Statistical Institute has highlighted that African immigrants represent about 1% of the Italian population. Moreover, they have the lowest level of education and they mainly work in low-qualified positions [22], thus depicting them as a low-status minority. Importantly, members of the Black minority group often interiorize these negative representations about their group as evidenced by their spontaneous responses [23]. For instance, Nosek, Banaji and Greenwald (2002) [24] collected online data from a wide sample of participants who performed an implicit race-bias test [i.e., IAT, 17] and showed that White people had a strong ingroup preference whereas Black people exhibited less polarized group preference [also see 14]. Based on this evidence, it could be expected group membership to exert an asymmetrical influence upon gaze cueing. Specifically, White participants should maximally differentiate the ingroup from the outgroup and thus exhibit a magnified gaze cueing effect when viewing a White face with respect to a Black face. In contrast, Black participants should show a similar gaze cueing effect independent of whether the gaze belongs to a White or a Black face.

In the following experiments we aimed at testing the two alternative scenarios described above. In particular, in Experiment 1, we presented White participants (i.e., the majority group) with a modified spatial cueing paradigm including pictures of White and Black individuals.

## Methods

### Experiment 1: the perspective of the majority group

#### Participants

Thirty-seven White Italian students (25 females) from the University of Padova participated in the study. Their mean age was 24 years. All participants provided a written informed consent prior to taking part in the experiment. Twenty participants took part in the study on a voluntary basis whereas the other seventeen were paid 10 euros for their participation. The experiment was conducted in accordance with the guidelines laid down in the Declaration of Helsinki, and participants were fully informed that their data would be analyzed anonymously.

#### Apparatus, Stimuli, and Procedure

Upon arrival in the lab, participants were greeted by a White experimenter. Presentation of the stimuli and registration of the responses were controlled by E-prime 1.1. Stimuli were presented on a 17″ monitor with a resolution of 1024×768 connected to an IBM compatible Pentium IV computer. The participants sat 57 cm from the computer monitor.

Sixteen avatar 3-D full-color faces created with FaceGen 3.1 software (2006) were used (4 Black females, 4 Black males, 4 White females, and 4 White males). Independent observers showed perfect agreement in the categorization of stimuli as representing either Black or White faces. For each face, the same software was used for creating a first image with direct gaze, a second image with averted gaze to the right and a third image with averted gaze to the left. Faces did not display additional characteristics such as hair or clothes. Each face subtended a visual angle of 16.8° in height and 14.4° in width. Faces presented to paid and unpaid participants were the same, except for the fact that those presented to paid participants were matched for luminance (2.5 cd/m^2^).

**Figure 1:**
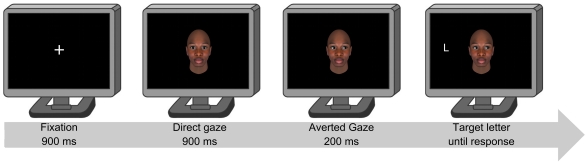
Schematic illustration of the sequence of events in Experiments 1, 2, 3 and 4. The Figure illustrates the experimental procedure within a single trial. Gaze direction was not informative as to target location. A cue-target spatially incongruent trial with a Black face is illustrated. Stimuli are not drawn to scale.

Each trial began with a white fixation point which remained on the screen for 900 ms, then a face with direct gaze appeared remaining on the screen for another 900 ms. Next, the image of the same face with gaze averted leftwards or rightwards was superimposed, thus conveying the impression of the eyes looking left or right. A target letter (either L or T) then appeared to the left or to the right of the face after 200 ms (see Figure 1). This short duration was employed in order to tap into exogenous processes [25].

Gaze direction was not informative with respect to target location and trial order was randomized. The target letter appeared at approximately 11° from the center of the screen, aligned with the horizontal meridian. The background color of the monitor was set to black and the target letter was set to white in 24-point Arial bold font. The specific color of the target is likely to be irrelevant, in line with previous research showing that chromatic features do not affect gaze cueing [26]. The participants were required to identify the target letter by pressing one of two labeled response keys of the keyboard, namely “d” and “k”, with their index fingers. The association between target letter and response key was counterbalanced across participants. The experiment comprised 256 trials. There were 64 trials for each level of cue-target spatial congruency and race of the cueing face. Before starting the experiment, participants were explicitly told that gaze direction was not informative as regards target location and they were instructed to maintain fixation at the center of the screen throughout a trial.

### Experiment 2: the perspective of the minority group

#### Participants

Thirty-two students (16 females) from the University of Padova who self-identified as Black individuals were paid 10 euros for their participation. They were all born in different countries of Sub-Saharan Africa. Their mean age was 26.5 years. The experiment was conducted in accordance with the guidelines laid down in the Declaration of Helsinki, and participants were fully informed that their data would be analyzed anonymously.

#### Apparatus, Stimuli, and Procedure

Apparatus, stimuli, and procedure were the same as those used in Experiment 1.

### Experiment 3: the role of the context

#### Participants

Seventy-two White Italian students (60 females) from the University of Padova participated in partial fulfillment of course requirements. Their mean age was 20 years. All participants provided a written informed consent prior to taking part in the experiment. The experiment was conducted in accordance with the guidelines laid down in the Declaration of Helsinki, and participants were fully informed that their data would be analyzed anonymously.

#### Apparatus, Stimuli, and Procedure

The apparatus, stimuli, and procedure were the same as in the previous experiments, with only one exception, namely the manipulation of an additional between-participants factor. Indeed, participants were randomly assigned to either a Mixed condition (N = 36) or a Blocked condition (N = 36). In the Mixed condition, we aimed at replicating the results obtained in Experiment 1. The procedure was exactly the same as in Experiment 1 (see Figure 1). Participants completed 256 trials divided into two blocks where Black and White faces could appear in a random order within each block. In the Blocked condition, participants completed 256 trials divided into two blocks with the race of the cueing face kept constant within each block. The relative order of the two blocks was counterbalanced across participants.

### Experiment 4: addressing the role of familiarity

#### Participants

Seventeen White Italian students (14 females) from the University of Padova took part in the experiment and were paid 10 euros for their participation. Their mean age was 23 years. All participants provided a written informed consent prior to taking part in the experiment. The experiment was conducted in accordance with the guidelines laid down in the Declaration of Helsinki, and participants were fully informed that their data would be analyzed anonymously.

#### Apparatus, Stimuli and Procedure

Apparatus and procedure were the same as in Experiments 1 and 2 (see Figure 1). Sixteen avatar 3-D faces created with FaceGen 3.1 software (2006) were used: Eight Caucasian White faces (4 females, 4 males) and eight multi-racial faces (4 females, 4 males). Multi-racial faces resulted from a balanced combination of faces belonging to different races (i.e., African, Caucasian, Asian). As in previous experiments, for each face, we created a first image with direct gaze, a second image with averted gaze to the right and a third image with averted gaze to the left. In order to accentuate the difference between the two sets of stimuli, and to resemble the different skin color of White and Black faces, we turned the color of multi-racial faces to a green shade, following a procedure similar to that used by Avenanti and colleagues (2010) [14]. This also enabled to decrease the familiarity with the stimuli. We reasoned that White participants had a better expertise in processing White over green multi-racial faces. If familiarity had a role in influencing gaze-mediated orienting, then we should have found greater gaze cueing effects for White faces as compared to green multi-racial faces. White and green faces were matched for luminance (2.5 cd/m^2^).

## Results

### Experiment 1

**Figure 2:**
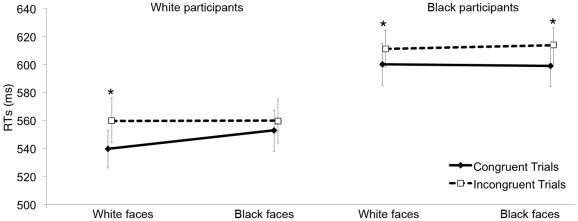
Gaze cueing effect in Experiment 1 and Experiment 2. Panel A shows participants' mean reaction times in Experiment 1 as a function of Congruency and Racial group membership. Panel B shows participants' mean reaction times in Experiment 2 as a function of Congruency and Racial group membership. The black line illustrates spatially congruent trials, the dashed line illustrates spatially incongruent trials. Bars represent SEM. *p<.05.

A 2 (cue-target spatial congruency: congruent vs. incongruent)×2 (racial group membership: White vs. Black)×2 (payment: paid vs. unpaid) ANOVA was performed on mean reaction times for correct responses. Participants were faster to identify the target when it appeared in the gazed-at location (i.e., spatially congruent trials, *M* = 546 ms, *SE* = 14) as compared to when it appeared in the opposite location (i.e., spatially incongruent trials, *M* = 560 ms, *SE* = 16), *F*(1,35) = 9.594, *p* = .004, η^2^_partial_ = .215. More importantly, this main effect was qualified by a significant Congruency×Racial group membership interaction, *F*(1,35) = 9.233, *p* = .004, η^2^_partial_ = .21, indicating that participants shifted their attention in response to the averted gaze of a White face, *t*(36) = −4.138, *p*<.001, but not in response to the averted gaze of a Black face, *t*(36) = −1.312, *p* = .198 (see Figure 2A). Importantly, neither the main effect of payment nor any interaction involving this factor were significant, all *p*s>.146, thus confirming that participants' performance was unaffected by the financial reward. Moreover, as anticipated earlier, the face stimuli presented to paid participants were matched for luminance, whereas face stimuli presented to unpaid participants were not. Because no significant difference emerged in the performance of the two samples, we can reasonably argue that the luminance of the stimuli did not play a relevant role in driving results.

An identical ANOVA was conducted on the percentage of errors (3.8%). No significant effect emerged, thus making the occurrence of any speed-accuracy tradeoff unlikely.

The present results show that racial group membership plays an important role in gaze cueing.

This pattern is even more remarkable given that it was obtained with a sample drawn from a population of undergraduate students who typically hold rather liberal social attitudes [27]. It might be expected that such modulation would be even magnified among more conservative respondents who hold more negative attitudes towards the outgroup and consider social hierarchies a natural aspect of social life [28].

The observed pattern cannot distinguish between the two scenarios illustrated in the Introduction. In Experiment 2, we explored the influence of racial group membership on gaze-mediated orienting of attention from the perspective of Black participants. Following the first hypothesis, we should observe a reversed pattern with respect to the one emerged in Experiment 1, that is Black participants should shift their attention only when the averted gaze belongs to the face of a Black individual. As discussed earlier, however, there is consistent evidence that minority group members interiorize the subordinate image associated with their group membership since their childhood [23], [29], and do not automatically prioritize their ingroup as much as White people do. Thus, in accordance with the second hypothesis, the magnitude of gaze cueing should be unaffected by the race of the face.

### Experiment 2

Data from one participant were excluded from analyses because more than 10% of his reaction times were above 1000 ms thus leaving thirty-one participants for the analyses.

A 2 (cue-target spatial congruency: congruent vs. incongruent)×2 (racial group membership: White vs. Black) repeated measures ANOVA was performed on mean reaction times for correct responses. Participants were faster to identify the target when it appeared in the gazed-at location (i.e., spatially congruent trials, *M* = 599 ms, *SE* = 14) as compared to when it appeared in the opposite location (i.e., spatially incongruent trials, *M* = 611 ms, *SE* = 13), *F*(1,30) = 10.477, *p* = .003, η^2^_partial_ = .259. The Congruency×Racial group membership interaction was not significant, *p* = .71, η^2^_partial_ = .005. To further ensure that there was no difference in the cueing effect according to the racial group membership of the cueing face, planned contrasts were conducted showing that participants shifted their attention in response to the averted gaze of both White, *t*(30) = −2.419, *p* = .022, and Black faces, *t*(30) = −2.326, *p* = .027 (see Figure 2B). An identical repeated measure ANOVA was conducted on the percentage of errors (2.2%). Only a significant main effect of Congruency emerged, *F*(1,30) = 5.091, *p* = .031, η^2^_partial_ = .145, showing that participants committed more errors on incongruent trials (*M* = 2.6%, *SE* = .6) than on congruent trials (*M* = 1.8%, *SE* = .4).

A further 2 (cue-target spatial congruency: congruent vs. incongruent)×2 (racial group membership: White vs. Black)×2 (Experiment: 1 vs. 2) mixed ANOVA was conducted on correct reaction times in order to compare the behavior of White and Black participants. The three way interaction was significant, *F*(1,66) = 4.052, *p* = .048, η^2^_partial_ = .058, confirming that the cueing effect exhibited by White and Black participants was differently affected by the racial group membership of the presented faces. Indeed, White participants (i.e., the majority group) showed a significant cueing effect towards White but not towards Black individuals, whereas Black participants (i.e., the minority group) showed a significant cueing effect towards both White and Black individuals.

Overall, the two experiments seem to indicate that social factors impact onto the cueing effect. In order to further support this interpretation, in Experiment 3, we employed a specific experimental manipulation that is known to shape the salience of social factors such as group membership. Indeed, research from the person perception domain has shown that the automaticity of category activation is modulated by the task environment, so that categorical knowledge is activated when participants are presented with exemplars belonging to two different categories (i.e., in mixed order), but not when stimuli are blocked according to their category [e.g.], [ 30, 31]. In the former condition, categorical membership is highly salient due to context-induced comparison processes, whereas in the latter case the distinctiveness of the exemplars is reduced by the presence of a homogeneous stimulus context. Therefore, in Experiment 3, we aimed to ascertain whether the modulation of gaze cueing observed in Experiment 1 was sensitive to whether White and Black faces were presented either intermixed or in separate blocks of trials. We expected to replicate the pattern of Experiment 1 when both White and Black faces were presented in a mixed order. In contrast, when stimuli were blocked by race, we expected White participants to exhibit a significant cueing effect for both White and Black faces, since the race of the faces was likely to be no longer salient given the absence of any term of comparison.

### Experiment 3

**Figure 3:**
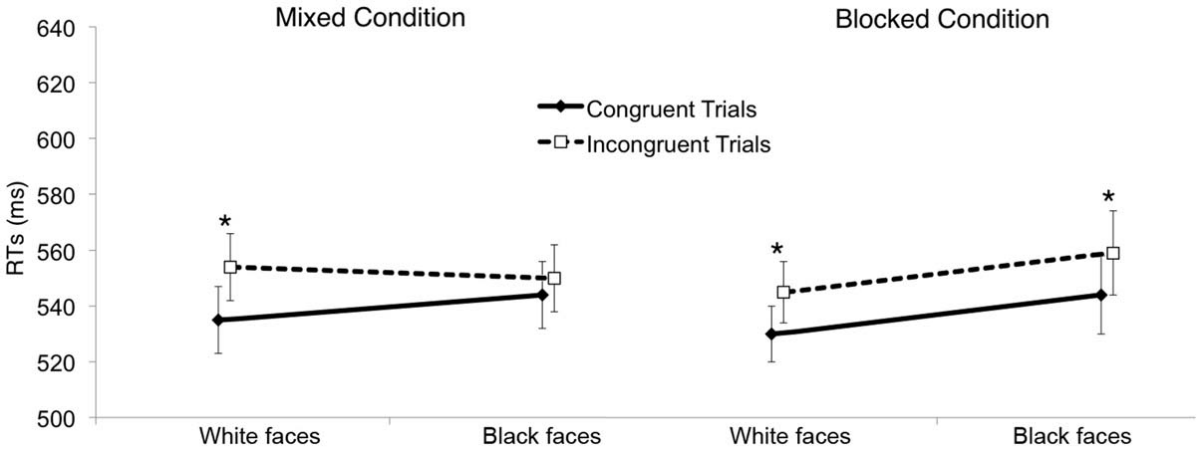
Gaze cueing effect in Experiment 3. Panel A shows mean reaction times for the Mixed Condition as a function of Congruency and Racial group membership. Panel B shows mean reaction times for the Blocked Condition as a function of Congruency and Racial group membership. The black line illustrates spatially congruent trials, the dashed line illustrates spatially incongruent trials. Bars represent SEM. *p<.05.

For completeness, a 2 (cue-target spatial congruency: congruent vs. incongruent)×2 (racial group membership: White vs. Black) repeated measure ANOVA with Condition (mixed vs. blocked) as a between participant factor was performed on mean reaction times for correct responses. The three-way interaction was not significant, *F*(1,70) = 2.912, *p* = .092, η^2^_partial_ = .040. However, given our strong a priori hypotheses, we submitted mean reaction times for correct responses to two identical 2 (cue-target spatial congruency: congruent vs. incongruent)×2 (racial group membership: White vs. Black) repeated measure ANOVAs separately for the two conditions: Mixed and Blocked. In the Mixed condition, we replicated the effect obtained in Experiment 1. A significant main effect of Congruency emerged, revealing that participants were faster to identify the target when it appeared in the congruent location (*M* = 540 ms, *SE* = 12) as compared to the incongruent location (*M* = 552 ms, *SE* = 12), *F*(1,35) = 12.849, *p* = .001, η^2^_partial_ = .269. Moreover, a significant Congruency×Racial group membership interaction emerged, *F*(1,35) = 4.255, *p* = .047, ^2^_partial_ = .108. Planned contrasts showed a significant cueing effect in response to White faces, *t*(35) = −4.616, *p*<.001, but not to Black faces, *t*(35) = −1.146, *p* = .260 (see Figure 3A). In the Blocked condition, a significant effect of Congruency emerged, in that participants were faster to detect the target when it appeared in the congruent (*M* = 537 ms, *SE* = 11) as compared to the incongruent location (*M* = 552 ms, *SE* = 12), *F*(1,35) = 28.670, *p* = .001, η^2^_partial_ = .450. Importantly, in this case the interaction between Congruency and Racial group membership was not significant, *p* = .896. Planned contrasts showed that the cueing effect was significant and of comparable magnitude independent of whether the cue was provided by a White, *t*(35) = −5.644, *p*<.001, or a Black face, *t*(35) = −3.358, *p* = .002 (see Figure 3B). Two identical repeated measure ANOVAs were conducted on the percentage of errors for the Mixed condition (2.2%) and the Blocked condition (2.1%). A significant effect of Congruency emerged only in the Blocked condition, *F*(1,35) = 7.785, *p* = .008, η^2^_partial_ = .182, indicating that participants made significantly more errors in the case of incongruent (*M* = 2.5%, *SE* = .4) as compared to congruent trials (*M* = 1.7%, *SE* = .3). Thus, no evidence for speed-accuracy tradeoff emerged (see File S1 of the Supporting Information for an additional explorative analysis).

These results show that, when White and Black faces were presented in a mixed order, White participants exhibited a gaze-cueing effect only in response to White faces. In contrast, when participants faced two different blocks in which race was invariant in a block, they shifted attention to every available cue and a cueing effect emerged in response to both White and Black faces. This pattern is taken as additional evidence that the modulation of gaze cueing observed in the previous experiments is related to social rather than low-level perceptual properties of the stimuli. In this regard, one may have argued that the absence of cueing effect exhibited by White participants for Black faces in Experiment 1 was simply due to the different physical properties (e.g., contrast between the skin and the sclera) of the faces, with faces of Black individuals eliciting a weaker perception of averted gaze than faces of White individuals. The observation that Black faces were able to trigger a cueing effect only under specific contextual circumstances allows us to rule out this alternative account and highlights that the broader experimental context can have a fundamental role in the emerging of the cueing effect, thus challenging the unconditioned automaticity of gaze-mediated orienting of attention [e.g.], [ 32]–[34].

So far, we have discussed our findings as reflecting socially relevant differences associated to racial group membership. However, one may argue that the effects obtained in the previous experiments were instead due to differences in perceptual familiarity. Indeed, it could be hypothesized that White participants have a perceptual advantage in processing White faces because of the familiarity they have with people of their racial ingroup. On the contrary, Black participants might be expected to be equally good at processing faces of both White individuals, who represent the majority of the population in their living environment, and Black individuals, who represent their direct relatives.

In order to investigate whether perceptual familiarity of the face stimuli could play a role in driving our results, we performed another experiment in which we presented participants with perceptually familiar and unfamiliar faces as stimuli in a gaze-cueing paradigm.

### Experiment 4

Data from two participants were excluded from analyses because more than 10% of their reaction times were above 1000 ms thus leaving fifteen participants for the analyses.

A 2 (cue-target spatial congruency: congruent vs. incongruent)×2 (familiarity: familiar vs. unfamiliar) repeated measures ANOVA was conducted on mean reaction times for correct responses. A significant effect of Congruency emerged, *F*(1,14) = 9.706, *p* = .008, η^2^_partial_ = .409, showing that participants were faster on congruent (*M* = 523 ms, *SE* = 18) as compared to incongruent trials (*M* = 533 ms, *SE* = 18). No other significant effect emerged, all *p*s>.46. A second ANOVA with the same factors as above was conducted on the percentage of errors (3.9%). A marginally significant effect of familiarity emerged, *F*(1,14) = 3.958, *p* = .067, η^2^_partial_ = .220, reflecting the tendency of participants in committing more errors when the face was familiar (i.e., White, *M* = 4.4% *SE* = 1.2), as compared to when it was unfamiliar (i.e., green multi-racial, *M* = 3.3% *SE* = .9). No other significant effect emerged, all *p*s>.137, which makes the possibility of a speed-accuracy trade-off unlikely.

The results of Experiment 4 demonstrate that a difference in the perceptual familiarity of the face stimuli is not sufficient to modulate the gaze-cueing effect. Indeed, even though it could be reasonably expected that White participants had a better expertise in processing White faces over green multi-racial faces, the cueing effect was not modulated by the nature (familiar vs. unfamiliar) of the cueing faces. This suggests that the results obtained in the previous experiments, in which Black and White faces were used, are unlikely to be driven by any eventual difference in perceptual familiarity associated to these two classes of stimuli.

## Discussion

In recent years, research has focused on the social side of gaze-mediated orienting of attention by investigating the interplay between this phenomenon and social information [7], [8], [11], [12], [35]. In the present set of experiments, we focused on a specific social aspect, namely racial group membership. We addressed this specific possible moderator at the light of previous evidence showing that several basic cognitive processes are highly sensitive to this factor. With regards to the specific phenomenon of gaze cueing, we reasoned that racial group membership might exert its influence in two alternative ways. On the one hand, one could have expected gaze cueing to be magnified for faces belonging to participants' ingroup, independent of the specific race. This scenario would be in line with the idea that the similarity between the respondent and the cueing face is the key factor underlying this modulation [15]. On the other hand, however, not all groups are alike and it is well known that, in most Western societies, Black people are perceived as a low-status minority compared to White people and that prejudice towards them is still highly rooted [21], [23], [24]. This holds true also in the social environment where the present experiments were carried out, namely the Italian context. In light of this reasoning, we hypothesized the enhanced gaze cueing effect for ingroup faces to emerge only for participants belonging to the majority group (i.e., White individuals). In contrast, for participants belonging to the minority group (i.e., Black individuals), the magnitude of the gaze cueing would be unaffected by the race membership of the face. Consistently with this latter scenario, the results of Experiments 1 and 2 showed that White participants exhibited a significant cueing effect only in response to the gaze of White faces, whereas Black participants exhibited a significant cueing effect in response to the gaze of both White and Black faces.

Although perceptual familiarity represented a potential alternative explanation for these findings, the results of Experiment 4 suggest that the different degree of perceptual familiarity with the face stimuli is not sufficient to modulate gaze-mediated orienting. Indeed, the cueing effect exhibited by participants in Experiment 4 was not modulated by the presentation of either a White Caucasian face or a green multi-racial face. Had perceptual familiarity played a relevant role, we should have observed a significantly reduced cueing effect for the unfamiliar green multi-racial faces, which clearly was not the case.

In light of the arguments discussed above, we feel confident that the effects emerged in Experiments 1 and 2 genuinely reflect the impact of race membership. In this regard, it is worth noting that, in the present experiments, we focused on overall intergroup differences, comparing responses towards White and Black individuals in the lack of any more specific information about the faces used as stimuli. However, when specific characteristics of a single individual are inconsistent with the social information associated to his/her group (e.g., the face of a well-known high-status Black person), it might be predicted that individualized information can override the effects of group membership, even for White respondents. In addition, it was hypothesized that group memberships have to be salient in the specific social context in order to exert a modulation. Experiment 3 was specifically designed to test whether the modulation of gaze cueing observed in Experiment 1 for White participants was sensitive to the context. The results showed that when the experimental context allowed for a comparison between Black and White faces (i.e., mixed condition), White faces were prioritized over Black faces. On the contrary, when Black and White faces were kept constant within two distinct blocks (i.e., blocked condition), they both drove gaze-mediated orienting. This result is important in that it shows that the modulation of gaze cueing as a function of race membership critically depends upon whether or not the context favored the activation of different social information related to different racial groups.

Overall, the results of the present set of experiments demonstrate that social information associated to racial group membership can affect orienting of attention. It is likely that the specific kind of modulation observed here (i.e., only White participants differentiate between White and Black faces) is not universal but it occurs each time the two groups are not associated to equally positive representations in terms of attributes and social status. In support of this view, a questionnaire administered to a sample of White and Black individuals from the same population as participants in the present experiments confirmed that Black people were considered as the minority group with respect to White people, independent of the race of the respondent (see File S2 for a detailed description of the questionnaire and the related statistical analyses). This pattern is further confirmed by research findings obtained with Black preschool-aged children in Italy who show pro-White biases similar to those expressed by White children [Castelli, unpublished data].

It could be argued that the modulation emerged in the present research reflects the impact of different factors related to the perception of a particular social group. Indeed, each social group is associated to different affective responses, evaluations, stereotypes and perceptions of social status, and it is difficult to identify which specific factors are involved in the observed modulation. In addition, these affective and cognitive aspects of attitudes towards the outgroup are so strictly interconnected that the identification of the unique impact of each of them is problematic. For instance, prominent models about intergroup perception [e.g., 36] suggest that social structural variables, such as perceived competition and status, do indeed shape the content of the stereotypes applied to the various social groups. In this sense, social status can be considered as a higher-order variable determining stereotypical views. Overall, in the cultural context addressed in the current research, social status emerged to be intrinsically linked to ethnic identity (see File S2). However, because status was not directly manipulated in the current experiments, we cannot exclude that other factors systematically associated to White and Black individuals may have affected our findings. We know that White and Black targets typically activate different affective reactions, namely more negative spontaneous responses towards Black people [37]. Whether differential evaluations can indeed modulate gaze-mediated orienting of attention, however, remains an open question. One could argue that when presented with faces associated to negative reactions that could thus be perceived as threatening, the focus of attention is restricted so that responses to the upcoming stimuli (e.g., lateralized targets) should be significantly slower overall. We did not find any main effect of the race of the faces, and thus the lack of cueing effect for White respondents in the case of the Black faces cannot be explained in terms of more difficult disengaging of attention from those faces. In addition, evidence has been reported that robust gaze cueing effects can be obtained even in the case of emotionally charged stimuli such as angry faces [e.g.], [ 38, 39]. Future studies, however, will have to more closely determine whether the valence of affective responses towards social targets can also play a role in the modulation of gaze cueing effects.

In sum, differences in perceived social status are a likely candidate factor for explaining the current findings. In this domain, a recent study on non-human primates investigated the role of status in modulating gaze-mediated orienting of attention [12]. In particular, the authors showed that high-status exemplars only follow the gaze of same status conspecifics whereas low-status exemplars follow the gaze of both higher and same status conspecifics. Shepherd et al. (2006) [12] argued that the modulation of the cueing effect by status exhibited by monkeys may be observable also in humans since monkeys and humans also share the basic processes of reflexive social attention [40]. It is well known that low-ranking animals monitor other monkeys in the group more frequently than high-ranking animals [41], [42], likely for avoiding conflict [43]. Similarly, it has recently been demonstrated in humans that high-status individuals are gazed at more often than low-status individuals [44]. Our results fit well with previous literature concerning the different nonverbal behavior that is exhibited by high- and low-status individuals [45]. Indeed, members of low-status groups are hypothesized to better monitor their environment and they tend to be more vigilant and guarded. Gaze-mediated orienting can be considered as a tool for successfully inspecting the environment [1]. Indeed, by following others' line of sight, it is possible to detect events of potentially shared interest. Thus, our findings may suggest that low-status individuals (i.e., Black individuals) are more likely to spend cognitive resources in monitoring the environment.

To conclude, we found that racial group membership modulates gaze-mediated orienting of attention in humans. All together, results obtained in the current set of experiments show that gaze-mediated orienting of attention is not an ubiquitous phenomenon which takes place every time an averted gaze is seen, but it depends on the kind of stimuli presented and on the social relationship between the observer and the person perceived.

## Supporting Information

File S1 **Additional explorative analysis of Experiment 3.**
(DOC)

File S2 **Description of the questionnaire and the related statistical analyses.**
(DOC)
