# Supplementary material for: Racial Group Membership Is Associated to Gaze-Mediated Orienting in Italy
Source: PLoS One. 2011 Oct 4;6(10):e25608. doi: 10.1371/journal.pone.0025608 (PMC3186779; doi:10.1371/journal.pone.0025608)
Supplement: File S1 — Additional explorative analysis of Experiment 3. (DOC) [file pone.0025608.s001.doc]

**S1: Additional explorative analysis of Experiment 3**

The results of Experiment 3 showed that, when Black and White faces were presented in a mixed order, White participants exhibited a significant gaze-cueing effect only in response to White faces. On the contrary, when Black and White faces were presented in a blocked order, White participants exhibited a significant cueing effect independent of the racial group membership of the face stimuli.

In an attempt to understand in more detail the absence of modulation emerged in the Blocked condition, we analyzed participants’ reaction times taking into account whether responses to Black faces were given during the first or the second block. The hypothesis was that a significant effect should be always observed for White faces. For Black faces, the prediction was to observe a effect when those faces were presented in the first block, whereas when they were presented in the second block the effect was expected to disappear in the initial trials, because of carry-over effects (i.e., the White faces seen in the previous block were still active in memory as a term of comparison).

To this aim, we pooled together data from the first half (trials 1–64) and the second half of each block (trials 65-128) and then computed the effect separately for each cluster. As shown in Table 1, the results of planned comparisons confirmed the hypotheses, suggesting that having been exposed to White faces in the previous block, was sufficient to activate a comparison context and thus Black faces did not initially trigger a significant effect. However, when White faces viewed in the previous block were likely no longer active in memory, a significant effect emerged, as can be seen in cluster 2.

| First block: Black faces | | | | Second-block: White faces | | | |
| --- | --- | --- | --- | --- | --- | --- | --- |
| Cluster 1 | | Cluster 2 | | Cluster 1 | | Cluster 2 | |
| 19 ms (10) | p=.06  η^2^_p_=.10 | 27 ms (8) | p=.002  η^2^_p_=.26 | 17 ms (6) | p=.013  η^2^_p_=.17 | 11 ms (5) | p=.043  η^2^_p_=.12 |
| First block: White faces | | | | Second block: Black faces | | | |
| Cluster 1 | | Cluster 2 | | Cluster 1 | | Cluster 2 | |
| 20 ms (6) | p=.004  η^2^_p_=.22 | 13 ms (5) | p=.026  η^2^_p_=.14 | 2 ms (10) | p=.876  η^2^_p_=.001 | 16 ms (8) | p=.05  η^2^_p_=.11 |

Table 1. effects (mean (SE)) in the blocked condition of Experiment 3 as a function of block order.
