# Supplementary material for: Racial Group Membership Is Associated to Gaze-Mediated Orienting in Italy
Source: PLoS One. 2011 Oct 4;6(10):e25608. doi: 10.1371/journal.pone.0025608 (PMC3186779; doi:10.1371/journal.pone.0025608)
Supplement: File S2 — Description of the questionnaire and the related statistical analyses. (DOC) [file pone.0025608.s002.doc]

**S2: Description of the questionnaire and the related statistical analyses**

In order to ensure that White and Black individuals were indeed perceived as being the majority and the minority group, respectively, a questionnaire was administered to sixteen White students and twelve Black students drawn from the same population as participants in Experiments 1 and 2. Participants were first asked to separately report their perception of Black and White people in Italy in relation to their power, likelihood of success in the job market, and overall social status. Responses were provided along 9-point scales. A series of repeated measures ANOVAs with race of the respondent as a between-participants factor and evaluated group as a within-participants factor showed that Black people were evaluated as less powerful (*M*_Black_ = 2.9, *SE*_Black_ = .3; *M*_White_ = 7.7, *SE*_White_ = .2), less likely to have success (*M*_Black_ = 4.6, *SE*_Black_ = .4; *M*_White_ = 7.6, *SE*_White_ = .2) and of lower social status (*M*_Black_ = 4.2, *SE*_Black_ = .3; *M*_White_ = 6.4, *SE*_White_ = .3) as compared to White people, all *F*(1,26) > 16.076, all *p*s <.001. A significant interaction between the two factors emerged for both perceived power and likelihood of success, showing that Black individuals were evaluated as being less likely to gain power and success by Black as compared to White respondents, all *F*(1,26) > 5.033; all *p*s <.034. A second section of the questionnaire assessed which of the two racial groups was perceived as more likely to earn more money, be protected by the law, and influence political decisions. Responses were provided along 9-point scales anchored from “definitely a White individual” to “definitely a Black individual” with 5 as the mid-point labeled “both individuals to the same extent”. Results from three separate univariate ANOVAs showed that White and Black participants answered in a very similar way. The effect of the race of the respondent was marginally significant when judging the relative influence on political decisions, *F*(1,26) = 3.776, *p* = .063, η^2^_partial_ = .127, showing that, even though both Black (*M* = 2.0, *SE* = .4) and White (*M* = 3.1, *SE* = .4) participants thought that White people were more likely to influence political decisions, responses of Black participants were more polarized. The effect of the race of the respondent was not significant either for protection by the law, *p* = .945, or for likelihood of earning more money, *p* = .164. More specifically, three one-sample t-tests comparing the mean score with the mid-point of the scale showed that Black individuals were evaluated as being less likely to earn money (*M* = 3.9, *SE =* .3), be protected by the law (*M* = 3.8, *SE =* .3), and influence political decisions (*M* = 2.6, *SE =* .3), all *p*s <.001. Overall, these results confirmed the idea that Black people are perceived as belonging to a social minority group as compared to White people who clearly emerged as a more dominant social group. Importantly, this representation of the social hierarchy was very similar among White and Black respondents.

Finally, we explored the perception of attractiveness of each face stimulus by asking participants to evaluate it along 7-point Likert scales. The mean evaluation of Black and White stimuli was computed and submitted to a 2 (race of the respondent) × 2 (race of the stimuli) repeated measures ANOVA. Only a main effect of the race of the stimuli emerged, *F*(1,26) = 20.971, *p* <. 001, η^2^_p_ =.446, showing that Black stimuli (*M* = 4.10, *SE* = .2) were perceived as more attractive than White stimuli (*M* = 3.37, *SE* = .2). No significant interaction was found (*p* > .974), therefore the two groups of respondents displayed a high level of agreement in their evaluation of the stimuli. This is inconsistent with the idea that perceived attractiveness can account for the differences between White and Black participants in the gaze-cueing task.

QUESTIONNAIRE

(TRANSLATED FROM THE ITALIAN VERSION)

1) Please think about White and Black people in Italy.

In your opinion, how easy is it for White people to reach high places in our society?

| Extremely difficult |  |  |  |  |  |  |  | Extremely easy |
| --- | --- | --- | --- | --- | --- | --- | --- | --- |
| 1 | 2 | 3 | 4 | 5 | 6 | 7 | 8 | 9 |

In your opinion, how easy is it for Black people to reach high places in our society?

| Extremely difficult |  |  |  |  |  |  |  | Extremely easy |
| --- | --- | --- | --- | --- | --- | --- | --- | --- |
| 1 | 2 | 3 | 4 | 5 | 6 | 7 | 8 | 9 |

2) Please think about White and Black people in Italy.

In your opinion, how easy is it for White people with a brilliant curriculum to achieve a successful career?

| Extremely difficult |  |  |  |  |  |  |  | Extremely easy |
| --- | --- | --- | --- | --- | --- | --- | --- | --- |
| 1 | 2 | 3 | 4 | 5 | 6 | 7 | 8 | 9 |

In your opinion, how easy is it for Black people with a brilliant curriculum to achieve a successful career?

| Extremely difficult |  |  |  |  |  |  |  | Extremely easy |
| --- | --- | --- | --- | --- | --- | --- | --- | --- |
| 1 | 2 | 3 | 4 | 5 | 6 | 7 | 8 | 9 |

3) Please think about White and Black people in Italy.

When doing the same job, in your opinion, who is likely to earn more money between a White and a Black individual?

| Definitely a White individual |  |  |  | Both individuals to the same extent |  |  |  | Definitely a Black individual |
| --- | --- | --- | --- | --- | --- | --- | --- | --- |
| 1 | 2 | 3 | 4 | 5 | 6 | 7 | 8 | 9 |

4) Please think about White and Black people in Italy.

In your opinion, who is more protected by the law between a White and a Black individual?

| Definitely a White individual |  |  |  | Both individuals to the same extent |  |  |  | Definitely a Black individual |
| --- | --- | --- | --- | --- | --- | --- | --- | --- |
| 1 | 2 | 3 | 4 | 5 | 6 | 7 | 8 | 9 |

5) Please think about White and Black people in Italy.

In your opinion, who is more likely to influence political decisions between a White and a Black individual?

| Definitely a White individual |  |  |  | Both individuals to the same extent |  |  |  | Definitely a Black individual |
| --- | --- | --- | --- | --- | --- | --- | --- | --- |
| 1 | 2 | 3 | 4 | 5 | 6 | 7 | 8 | 9 |

6) Please think about White and Black people in Italy.

How would you rate the social status of White people?

| Extremely low |  |  |  |  |  |  |  | Extremely high |
| --- | --- | --- | --- | --- | --- | --- | --- | --- |
| 1 | 2 | 3 | 4 | 5 | 6 | 7 | 8 | 9 |

How would you rate the social status of Black people?

| Extremely low |  |  |  |  |  |  |  | Extremely high |
| --- | --- | --- | --- | --- | --- | --- | --- | --- |
| 1 | 2 | 3 | 4 | 5 | 6 | 7 | 8 | 9 |

ATTRACTIVENESS

Please indicate how attractive you judge each face using a scale from 1 to 7.

[Each face stimulus appeared here]

| Very unattractive |  |  |  |  |  | Very attractive |
| --- | --- | --- | --- | --- | --- | --- |
| 1 | 2 | 3 | 4 | 5 | 6 | 7 |
